# Genetic Diversification and Dispersal of Taro (*Colocasia esculenta* (L.) Schott)

**DOI:** 10.1371/journal.pone.0157712

**Published:** 2016-06-17

**Authors:** H. Chaïr, R. E. Traore, M. F. Duval, R. Rivallan, A. Mukherjee, L. M. Aboagye, W. J. Van Rensburg, V. Andrianavalona, M. A. A. Pinheiro de Carvalho, F. Saborio, M. Sri Prana, B. Komolong, F. Lawac, V. Lebot

**Affiliations:** 1 CIRAD, UMR AGAP, Montpellier, France; 2 Université de Ouagadougou, UFR-SVT, Ouagadougou, Burkina Faso; 3 CTCRI, Thiruvananthapuram, Kerala, India; 4 CSRI, Accra, Ghana; 5 ARC, Pretoria, South Africa; 6 FOFIFA, Antananarivo, Madagascar; 7 BG ISOPlexis, University of Madeira, Funchal, Portugal; 8 Universidad de Costa Rica, San Jose, Costa Rica; 9 LIPI, Bogor, West Java, Indonesia; 10 NARI, LAE, Morobe Province, Papua New Guinea; 11 VARTC, Santo, Vanuatu; 12 CIRAD, UMR AGAP, Port Vila, Vanuatu; National Cheng-Kung University, TAIWAN

## Abstract

Taro (*Colocasia esculenta* (L.) Schott) is widely distributed in tropical and sub-tropical areas. However, its origin, diversification and dispersal remain unclear. While taro genetic diversity has been documented at the country and regional levels in Asia and the Pacific, few reports are available from Americas and Africa where it has been introduced through human migrations. We used eleven microsatellite markers to investigate the diversity and diversification of taro accessions from nineteen countries in Asia, the Pacific, Africa and America. The highest genetic diversity and number of private alleles were observed in Asian accessions, mainly from India. While taro has been diversified in Asia and the Pacific mostly via sexual reproduction, clonal reproduction with mutation appeared predominant in African and American countries investigated. Bayesian clustering revealed a first genetic group of diploids from the Asia-Pacific region and to a second diploid-triploid group mainly from India. Admixed cultivars between the two genetic pools were also found. In West Africa, most cultivars were found to have originated from India. Only one multi-locus lineage was assigned to the Asian pool, while cultivars in Madagascar originated from India and Indonesia. The South African cultivars shared lineages with Japan. The Caribbean Islands cultivars were found to have originated from the Pacific, while in Costa Rica they were from India or admixed between Indian and Asian groups. Taro dispersal in the different areas of Africa and America is thus discussed in the light of available records of voyages and settlements.

## Introduction

Taro (*Colocasia esculenta* (L.) Schott) belongs to the family Araceae and is a highly polymorphic species [1]. This widely distributed crop is a staple food important in many localities in the humid tropics and subtropics. Taro extends to the temperate zones of East Asia, southern Africa, Australia and New Zealand [2]. There is a diversity of cultivars adapted to a range of microenvironments, from swidden fields, rain-fed upland and home gardens, to paddy fields and swamps in China [2], India [3], Vanuatu [4], Guadeloupe [5] and elsewhere. Taro is also referred to as *dasheen*, *eddoes*, *malanga* and *cocoyam* in the Caribbean and West Africa [2]. Like most other root and tuber crops, taro is vegetatively propagated, although seed production is possible. Natural breeding and population spread have been reported for wild taro [6]. Cultivars are propagated through the use of corms, cormels (also known as suckers), while vegetative propagation occurs through stolons in the wild [7].

Taro is probably one of the world’s oldest crops [4]. Archaeological studies indicate its usage as early as 28,000 years ago in the Solomon Islands [8]. Residue analyses of starch granules indicate that taro was already being processed during the early and mid-Holocene, at least 10,200 calibrated years before present (cal BP), on the wetland margins at Kuk Swamp in the highlands of Papua New Guinea (PNG) [9]. Taro was also cultivated by early settlers from 3,050 to 2,500 BP in the Pacific islands, as revealed by dating of starch grains from Bourewa, South West Viti Levu, Fiji [10]. Taro center of origin is still unresolved. Research continues to elucidate the centers of origin of this global crop, with northeast India and New Guinea being potential separate centers of domestication [2, 11, 12]. The greatest diversity of wild *Colocasia* species appears to extend from northeast India to southern China, within the Himalayan region of mainland Southeast Asia [13]. If wild taro populations were widespread before its cultivation began, then the taro cultivated today may have diverse and independent origins [13]. Based on genetic analyses, it could have been domesticated several times in different locations over a vast area ranging from India to South China, Melanesia and northern Australia [4].

The dispersal history of taro has been poorly studied in comparison with other crops such as sweet potato [14], banana [15] or jatropha [16]. Consequently, the dissemination of selected genotypes has not been documented. Taro is nowadays cultivated in Africa, where it has gained high importance mainly in Cameroon, Nigeria, Ghana and Burkina Faso [17, 18]. The period when taro spread to West Africa is unknown. It has been reported that taro was well established in Senegambia by AD 1500, long before Portuguese navigators reached West Africa [19]. It is also cultivated in America, especially in the Caribbean area. In the West Indies, taro was clearly mentioned in 1897 [20], but was probably introduced as a crop in Guadeloupe earlier during colonial times. The historian Du Terte in 1667 mentioned “fausse racine de Chine” (false Chinese root) but did not provide enough details to definitely confirm that he was referring to taro [5]. Taro is found on all Caribbean islands, in Central and South America and also in the US where it has been described as an invasive crop [21].

*Colocasia esculenta* has a basic chromosome number of 14 and two cytotypes: diploid with 28 chromosomes and triploid with 42 chromosomes [12]. Diploid cultivars are fertile while triploids are sterile. Cytogenetic, morphological and biochemical studies indicate that triploids may have originated as a result of autopolyploidy [22]. Kreike et al. [1] found diploids and triploids in Asia while all accessions analysed from the Pacific were diploids. In northeastern India, where diploids and triploids are found, the triploids seem to have evolved in response to the climatic conditions on the hills [11]. Several studies have attempted to describe taro genetic diversity using isozymes [23] and RAPD markers [24] and found the highest diversity in Indonesia. Later, isozymes [25] and AFLP [1] markers were used to analyze the genetic diversity of accessions from Vietnam, Thailand, Malaysia, Indonesia, the Philippines, Papua New Guinea and Vanuatu and highlighted two centers of secondary domestication. The first one is found in Southeast Asia and the second in Melanesia. Other studies have been conducted at the country level in Vanuatu [26], Papua New Guinea [27], India [28], Cuba [29] and Brazil [30]. However, no worldwide analysis has ever been conducted with African and American genotypes to characterize the diversity of taro and its dispersal patterns.

The overall objective of this study was to assess the genetic diversity of taro in countries located in areas considered as primary and secondary domestication centers for this species and in countries where it has been more recently introduced. In particular, we sought to determine: i- if the genetic diversity in Africa and America is comparable to that in Asia and the Pacific, ii- the mechanisms by which taro has diversified in Africa and America, namely by clonal reproduction with mutation or sexual reproduction, or both, and iii- if some cultivars have spread more than others. Finally, the distribution of taro is discussed on the basis of a combination of historical and linguistic data and our genetic data.

## Material and Methods

### Ethics statement

This work was conducted within the framework of the Europe-Aid project DCI-FOOD/2010/230-267 SPC “Adapting clonally propagated crops to climatic and commercial changes”. Partners of this project operate under the auspices of the International Network for Edible Aroids (INEA - www.ediblearoids.org), a cooperative network of countries established in April 2011, whereby edible aroids are used as a model to improve clonally propagated root and tuber crops of tropical countries. A majority of the partners in the network are signatories of the FAO International Treaty on Plant Genetic Resources for Food and Agriculture (ITPGRFA), and have agreed to share taro germplasm under the ITPGRFA Multilateral System of Access and Benefit‐sharing by Contracting Parties. The genetic diversity assessment was entrusted to CIRAD (Centre of International cooperation in Agronomical Research for Development) as part of the “Genetic Studies” workpackage of the project. Consequently, each partner conducted sampling in its own country and dried leaves were sent to CIRAD for genotyping. All partners have a national mandate for the collection and conservation of taro genetic resources and documentation of accompanying information. Samples were shipped to CIRAD according to the ITPGRFA guidelines using the Standard Material Transfer Agreement (SMTA).

### Plant material

An international core sample, representative of the cultivars cultivated from each country, was assembled to assess taro genetic diversity. Selected accessions corresponding to the most widespread cultivars in each participating country were collected. Hereafter, the word ‘cultivar’ refers to all plants sharing the same vernacular name and recognized by farmers as representing a distinct morphotype: i.e. all taro plants managed together by farmers and recognized as one entity at the community level. In this study, one accession of each cultivar was provided. A total of 321 cultivars were analyzed, comprising 64 from Asia, 196 from Africa, including 5 cultivars from Madeira, 19 from America and 42 from the Pacific region. Within the Vanuatu cultivars, seven were originated from Asia or were breeding lines between Asian and Pacific germplasm. The unbalanced number of cultivars was mainly due to geographical variation in the importance of taro. To complement the 321 cultivars received, 36 cultivars from the TANSAO (Taro Network for South East Asia and Oceania) core sample with known ploidy levels [1], maintained in the field at the Vanuatu Agronomical Research and Technical Centre (VARTC), were also included. These 36 cultivars were from Japan, the Philippines, Malaysia, Indonesia, Thailand and Vietnam. The ploidy level of cultivars collected *in situ* could not be determined. However, the ploidy levels of the 43 cultivars from India and 7 from Burkina Faso were determined by chromosome counting and flow cytometry, respectively. Among the 50 cultivars, 12 were diploid (n = 24 chromosomes) and 38 were triploid (n = 36 chromosomes). Overall, 357 cultivars were analyzed (S1 Table).

### DNA extraction and microsatellite amplification

Genomic DNA was extracted from 150 mg of dried leaves according to the protocol described by Risterucci et al. [31]. The quality and quantity of the extracted DNA was verified on 1% TBE agarose gels. All DNA extracts were stored at -20°C. A total of 64 microsatellite primer pairs developed from *Colocasia esculenta* [32–34] and *Amorphophallus paeoniifolius* [35] were screened and tested. Among the 64 microsatellite primer pairs, 11 were chosen and used in this study based on their reliable amplification profiles, high polymorphism and the ease with which the results could be unambiguously read and scored (S2 Table). The M13-tails added to forward primers for each microsatellite marker were labeled with IRD700 or IRD800 fluorochromes. Polymerase chain reactions (PCR) were carried out in a 10 μL reaction mix containing 25 ng of template DNA, 1 μl PCR buffer (10 μM Tris, 100 μM KCl, and 0.05% of glycerol), 1 U of *Taq* DNA polymerase (Life Technologies, USA), 2 mM MgCl_2_, 200 μM dNTPs and 0.1 μM of forward and reverse primers and M13 tail [36]. PCR cycling conditions were as follows: 5 min initial denaturation at 95°C, 10 amplification cycles with the shutdown method (-0.5°C per cycle) [45 s at 94°C, 1 min at Ta + 5°C, 1 min at 72°C], 25 amplification cycles [45 s at 94°C, 1 min at Ta, 1 min at 72°C], and a final 4 min elongation at 72°C. PCR amplifications were performed on PTC-100 thermocyclers (MJ Research) and genotyping was carried out on an IR2-DNA analyser (LiCor 4300 Sequencer). Due to polyploidy, AFLP Quantar Pro 1.0 software was used for automated data collection and to determine allele sizes. A double-blind reading was carried out by two different investigators and gels were rescored when there were discrepancies. Two control samples per microsatellite primer pairs were used. Each control sample was a bulk sample from three different individuals.

### Data analysis

Due to the ploidy of the studied species, partial heterozygosity makes it impossible to score genotypes exactly [37] because of difficulties in assigning the correct allele dosage for each locus and individual [38]. Alleles were then encoded as presence (1)/absence (0) data and co-dominant microsatellites were therefore scored as dominant. Due to these deviations from diploid meiotic behavior, indices such as expected heterozygosity (*He*), could not be used to study the genetic diversity of *C*. *esculenta* [39]. The resulting data matrix was thus used to calculate the following genetic parameters: total number of alleles (*An*), average number of effective alleles (*Ae'*), number of private alleles (*Ap*), Shannon information index (*I*) and genetic diversity (*μh*) for each country, when the number of cultivars was over five, and for each continent using the GenAlex 6.5 software [40].

Dissimilarities between all pairs of cultivars were estimated based on the Dice distance [41] and a minimum of 80% of valid data was required for each unit pair. An unrooted neighbor-joining tree was constructed using Darwin V5 software [42].

Taro is mainly vegetatively propagated crop; therefore clones were expected within our sample. One clone (genet) may include several cultivars (ramets). After genotyping, each cultivar is represented by a multilocus genotype (MLG). Several cultivars (ramets) can share the same MLG (genet) or differ by few mutations because of biological or methodological reasons such as somatic mutations, scoring errors and PCR artefacts. They are then assigned to the same clonal lineage or multilocus lineage (MLL). GENOTYPE software was first used in order to determine whether small genotypic differences between MLGs were a consequence of sexual recombination or somatic mutation, by calculating frequency distribution of distances among pairs of cultivars [43]. The histogram showed a valley between the first peak corresponding to the cultivars sharing the same MLG or those which differ by very few mutations and the second peak corresponding to the distances between cultivars derived from independent sexual reproduction events. Such valley was then used as reliable indicator for the threshold distance required to assign MLGs into distinct (MLLs) [44]. The MLGs grouped together, below the given threshold of difference, were assigned to distinct MLLs, while distinct MLGs above the threshold, were considered as unique genotypes (UG).

A second data set was obtained by keeping all UGs and only one representative for each MLL in each country. Consequently, some MLLs were shared between countries. For each country (except Nigeria and Trinidad & Tobago for which only one genotype was analyzed each), the index of clonal diversity (R) was estimated by R = (G-1)/(N-1), where G is the number of genotypes *ie* MLLs and unique genotypes (UG) in the sample and N is the number of cultivars analyzed. This index ranges from 0 (when all different samples analyzed correspond to a single MLL) to 1 (for a monoclonal stand) [45].

Within a clonal lineage, in order to visualize the relationship between MLGS and their distribution between countries, a network of each MLL was built. Within the same MLL, and for each locus, two MLGs differed by a maximum of one allele. We thus proceeded as described by Scarcelli et al. [46]. Diploid and triploid MLGs were reduced to haploid genotypes by eliminating at each locus, the allele shared by all MLGs within the MLL. Then relationships between MLGs within a lineage were inferred from a minimum spanning network (MSN) made using the software Haplophyle (www.haplophyle.cirad.fr).

An unrooted neighbor-joining tree was constructed with one representative of each MLL and all unique genotypes using Darwin V5 software [42]. The genetic structure was further explored using the Bayesian clustering algorithm implemented in STRUCTURE version 2.1 [47]. To perform this analysis, MLLs shared between countries were removed from the analysis and only a single copy of each discriminated MLL was retained in the whole dataset. The program was given no prior information on ancestral populations, and was run 20 times for each K ancestral population value, with *K* ranging from 1 to 10, under the admixture model, using a burn-in of 500,000 iterations and 1,000,000 Markov chain Monte Carlo iterations. We evaluated the inference of K using the *ad hoc* statistic Δ*K* method [48], as implemented in Structure Harvester software [49]. We assigned each individual to a group when the average proportion of membership was over 80% ancestry to their own cluster. In all groups, genotypes with membership probabilities under 80% were considered to be of ‘mixed origin’. After Bayesian clustering analysis, populations were redefined according to the results obtained. Genetic diversity parameters *(An)*, *(Ae')*, *(Ap)*, *(I)* and *(μh)* for each defined cluster were calculated as described above. We separately ran a new STRUCTURE analyses on each cluster in order to identify any sub-clustering within each cluster.

## Results

### Ploidy levels in *Colocasia esculenta*

Genotyping with 11 microsatellite marker loci revealed that the cultivars were partitioned into two distinct groups: one showing two alleles at all investigated loci and the second with up to three alleles at certain loci. If the ploidy level of cultivars bearing three alleles at least at one locus can be considered as triploid, the diploid status of cultivars with two alleles at all loci could not be assessed. Yet we obtained complete match between the number of alleles obtained after genotyping and the ploidy level of the cultivars checked from India, Burkina Faso and TANSAO (S1 Table). So, the ploidy level was inferred from the maximum number of alleles at all loci investigated. The number of cultivars showing three alleles at least at one locus differed among countries. All 56 cultivars from South Africa showed three alleles at least at one locus. The 42 cultivars from the Pacific region did not show more than two alleles. Also, cultivars from Caribbean Islands and Philippines did not present more than two alleles per locus, but this result should be taken with caution since the number of cultivars analysed was very low (one to three). (Table 1).

**Table 1:** Maximum number of alleles at loci, genetic diversity parameters and index of clonal diversity within the 357 cultivars of *Colocasia esculenta* obtained by genotyping with 11 nuclear microsatellite loci.

Continent	Countries	N	N with 2 alleles	N with 3 alleles	*An*	*Ae'*	*Ap*	*I*	*uh*	N° of MLL & UG	G	R
Africa	South Africa	56	0	56	33	1.03	2	0.04	0.02	2 MLL	2	0.02
	Burkina Faso	39	13	26	38	1.12	0	0.09	0.07	2 MLL	2	0.03
	Ghana	80	72	8	46	1.03	0	0.05	0.03	3 MLL	3	0.03
	Madagascar	12	10	2	51	1.1	5	0.11	0.07	5 (2 MLL & 3 UG)	5	0.36
	Réunion	3	2	1	NA	NA	NA	NA	NA	2 MLL	2	0.50
	Nigeria	1	0	1	NA	NA	NA	NA	NA	1 MLL	1	
	Madeira	5	1	4	49	1.16	0	0.14	0.12	5 (1 MLL & 4 UG)	5	1
	Total Africa	196	98	98	91	1.15	7	0.13	0.09		20	0.10
America	Costa Rica	5	1	4	44	1.13	1	0.12	0.1	3 (2 MLL & 1 UG)	3	0.50
	Martinique	10	10	0	30	1.07	1	0.06	0.05	3 (2 MLL & 1 UG)	3	0.22
	Guadeloupe	3	3	0	NA	NA	NA	NA	NA	3 (2 MLL & 1 UG)	3	1
	Trinidad & Tobago	1	1	0	NA	NA	NA	NA	NA	1 MLL	1	
	Total America	19	15	4	56	1.12	2	0.12	0.08		10	0.50
Asia	India	43	12	31	125	1.2	25	0.21	0.13	37 (5 MLL & 32 UG)	37	0.86
	Indonesia	37	33	4	109	1.16	7	0.17	0.11	31 (7 MLL & 24 UG)	31	0.83
	Philippines	11	11	0	51	1.11	2	0.11	0.08	10 (3 MLL & 7 UG)	10	0.90
	Japan	2	0	2	NA	NA	NA	NA	NA	2 MLL	2	1
	Malaysia	2	2	0	NA	NA	NA	NA	NA	2 UG	2	1
	Thailand	2	2	0	NA	NA	NA	NA	NA	2 (1 MLL & 1 UG)	2	1
	Vietnam	3	2	1	NA	NA	NA	NA	NA	3 (1 MLL & 2 UG)	3	1
	Total Asia	100	62	38	155	1.18	46	0.21	0.13		87	0.87
Pacific	PNG	11	11	0	56	1.11	4	0.12	0.08	11 UG	11	1
	Vanuatu	31	31	0	78	1.13	5	0.14	0.09	31 (1 MLL & 30 UG)	31	1
	Total Pacific	42	392	0	91	1.13	9	0.14	0.09		42	1

*N* number of cultivars collected in each country and continent, N with 2 alleles, number of cultivars showing two alleles at all loci investigated, N with 3 alleles, number of cultivars showing three alleles at least at one locus among the 11 investigated, *A*_*n*_ total number of alleles, *A*_*e’*_ number of effective alleles, *A*_*P*_ number of private alleles, *I* Shannon's information index, *μh* unbiased diversity, NA: non-analysed cultivars (due to the reduced number of cultivars within groups), N° of MLL & UG, number of MLLs and UGs within the country and continent, G, number of genotypes considered to calculate R, R, index of clonal diversity.

### Genetic diversity

A total of 195 alleles were amplified from the 11 microsatellite loci in the 357 cultivars. All loci were found to be highly polymorphic. The number of alleles observed in the whole dataset ranged from four to 31 alleles per locus (S2 Table). Genetic diversity parameters were calculated for the 12 countries, with at least five cultivars analysed. The number of alleles (*A*_*R*_), private alleles (*A*_*P*_), average number of effective alleles (*A*_*e’*_), Shannon information index (*I*) and genetic diversity (*μh*) are shown in Table 1. The highest numbers of alleles and private alleles were observed in cultivars from India (*A*_*p*_ = 25). Cultivars from Burkina Faso, Ghana and Madeira did not present any private allele, while those from Costa Rica, South Africa and Martinique had one to two private alleles. The *A*_*p*_ for the remaining countries from Asia and the Pacific ranged from four to seven. The Shannon Index and genetic diversity followed the same pattern, with India having the highest (*I)* and (*μh*), while South Africa and Ghana had the lowest. At the continent level, cultivars from Asia had the highest number of private alleles and genetic diversity while those from Africa, America and the Pacific had lowest genetic diversity parameter values.

Using the Dice matrix, the NJ unrooted tree showed structuring according to the countries and a high level of clonality (S1 Fig). Consequently, the number of MLGs was determined in the 357 cultivars. A total of 178 distinct MLGs were identified. The frequency distribution of genetic distances among pairs of MLGs gave bimodal histograms (S2 Fig). The threshold between the first peak corresponding to the cultivars sharing the same MLG or those which differ by very few mutations and the second peak corresponding to the distances between cultivars derived from independent sexual reproduction events was set to 8. Thus 118 MLGs were distinct and considered as unique genotypes (UG) and 42 closely related MLGs were grouped in 18 MLLs (Table 1). The highest indices of clonal diversity (R) were obtained for cultivars from Pacific countries, PNG and Vanuatu, with 1 each, followed by those from Asian countries, namely India and Indonesia, with 0.86 and 0.83, respectively. Cultivars from African countries (South Africa, Burkina Faso and Ghana) showed the lowest index of clonal diversity, ranging from 0.02 to 0.03. Even though 39, 80 and 56 cultivars from Burkina Faso, Ghana and South Africa were analyzed respectively, only two, three and two MLLs were identified, with two MLLs being shared between Burkina Faso and Ghana. Cultivars from Madeira presented 100 of index of clonal diversity. Cultivars from American countries had R ranging from 0.22 to 0.5. Guadeloupe showed R = 1, but only three cultivars were analyzed. The index of clonal diversity of TANSAO cultivars calculated per country revealed that those from Malaysia, Thailand, Vietnam and Japan had R = 1. TANSAO cultivars from Indonesia (16 cultivars) and the Philippines (11 cultivars) had R = 0.73 and 0.9, respectively. At the continent level, the highest index of clonal diversity was recorded for cultivars from the Pacific and Asia, while the lowest was obtained for those from Africa (0.1) and America (0.5).

**Fig 1:**
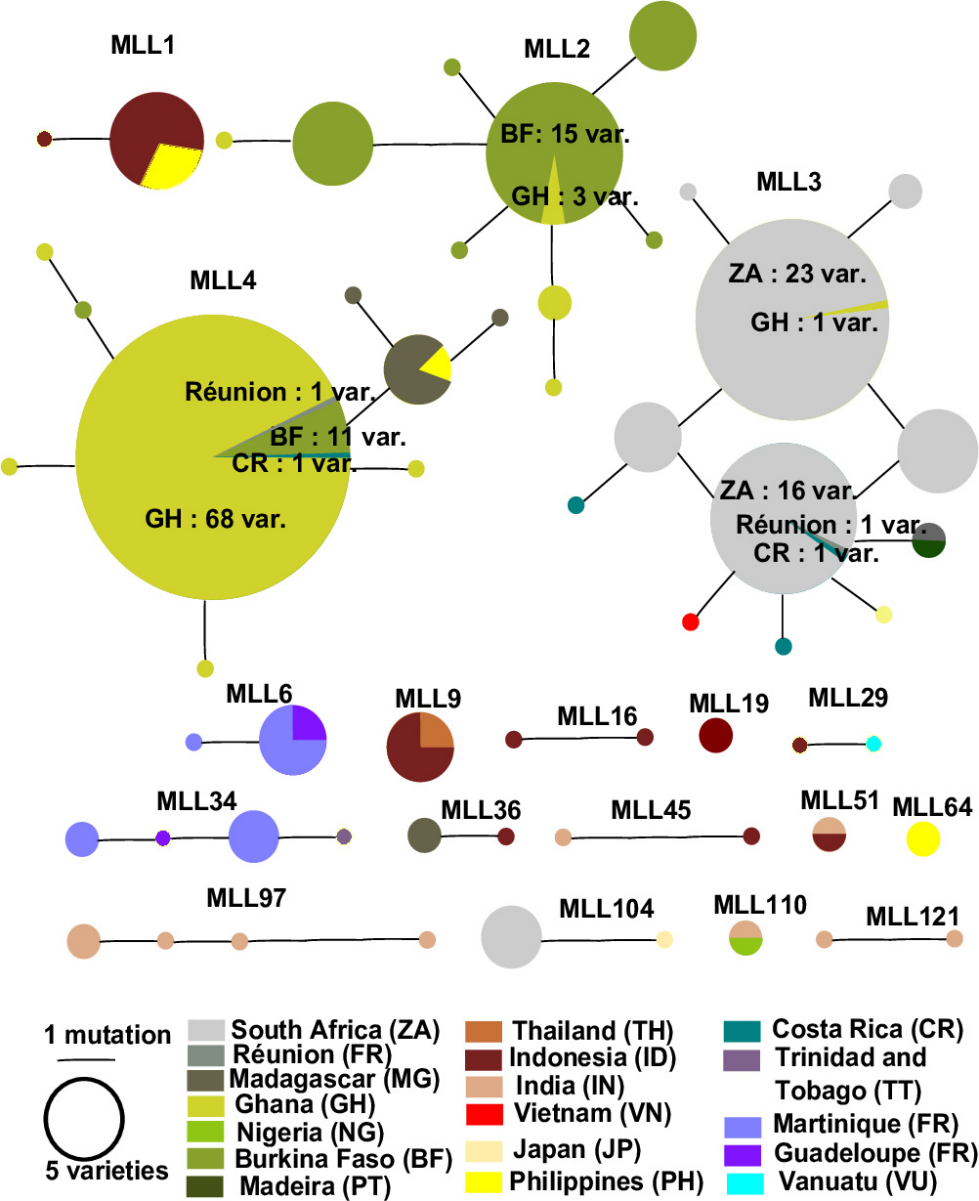
Minimum Spanning Network (MSN) representing the relationships between genotypes within the 18 multilocus lineages. Each country is represented by different colour. The size of each circle is proportional to the number of cultivars, except for MLLs 2, 3 and 4. Due to the high number of cultivars, the central pie chart for MLLs 2, 3 and 4 has been shown at half-size and the full number of cultivars contributing is shown.

Eighteen MLLs were identified in our sample. Three MLLs (2, 3 and 4) are substantially the most common in the data set. Within most MLLs, genotypes differed by only one allele. They were present either within one country or shared between different countries (Fig 1). Eleven MLLs were shared mostly between two countries, while two MLLs (3 and 4) were respectively shared between seven and six countries from Asia, Africa, and America (Table 2). They seem to have spread more widely. The country that had the highest number of MLLs shared with other countries was Indonesia, followed by India. PNG had no MLL shared with other countries, which may have been mainly related to the sampling bias *ie* no closely related genotypes were sampled. In Vanuatu, only one MLL was found to be shared with Indonesia. Five MLLs were shared between neighbouring countries (MLL 2, 6, 34, 45 and 51). Two MLLs (45 and 51) were only shared between India and Indonesia. Within MLL45, the two MLGs shared the same alleles except that the cultivar from Indonesia was diploid [1], while that from India was triploid (checked by chromosome counting) with one more allele (256) at locus CES-1A06. India, Indonesia and Philippines hosted each MLLs not shared with other countries: (MLL 16 and 19), (MLL 97 and121) and MLL64 respectively. MLLs 19 and 64 contained each two clonemates.

**Table 2:** Geographical distribution of the 18 multilocus lineages (MLLs) found within or in more than one country. Total number of cultivars for each MLL are shown below.

MLL number	1	2	3	4	6	9	16	19	29	34	36	45	51	64	97	104	110	121
Indonesia	3					3	2	2	1			1	1					
India											1	1	1		6		1	2
Philippines	1			1										2				
Thailand						1												
Japan			1													1		
Vietnam			1															
Vanuatu									1									
Madeira			1															
Madagascar				7							2							
Réunion			1	2														
South Africa			52													4		
Burkina Faso		26		13														
Ghana		7	1	72														
Nigeria																	1	
Trinidad and Tobago										1								
Guadeloupe					1					1								
Martinique					4					5								
Costa Rica			3	1														
Total	4	33	60	96	5	4	2	2	2	7	3	2	2	2	6	5	2	2

### Genetic structure

**Fig 2:**
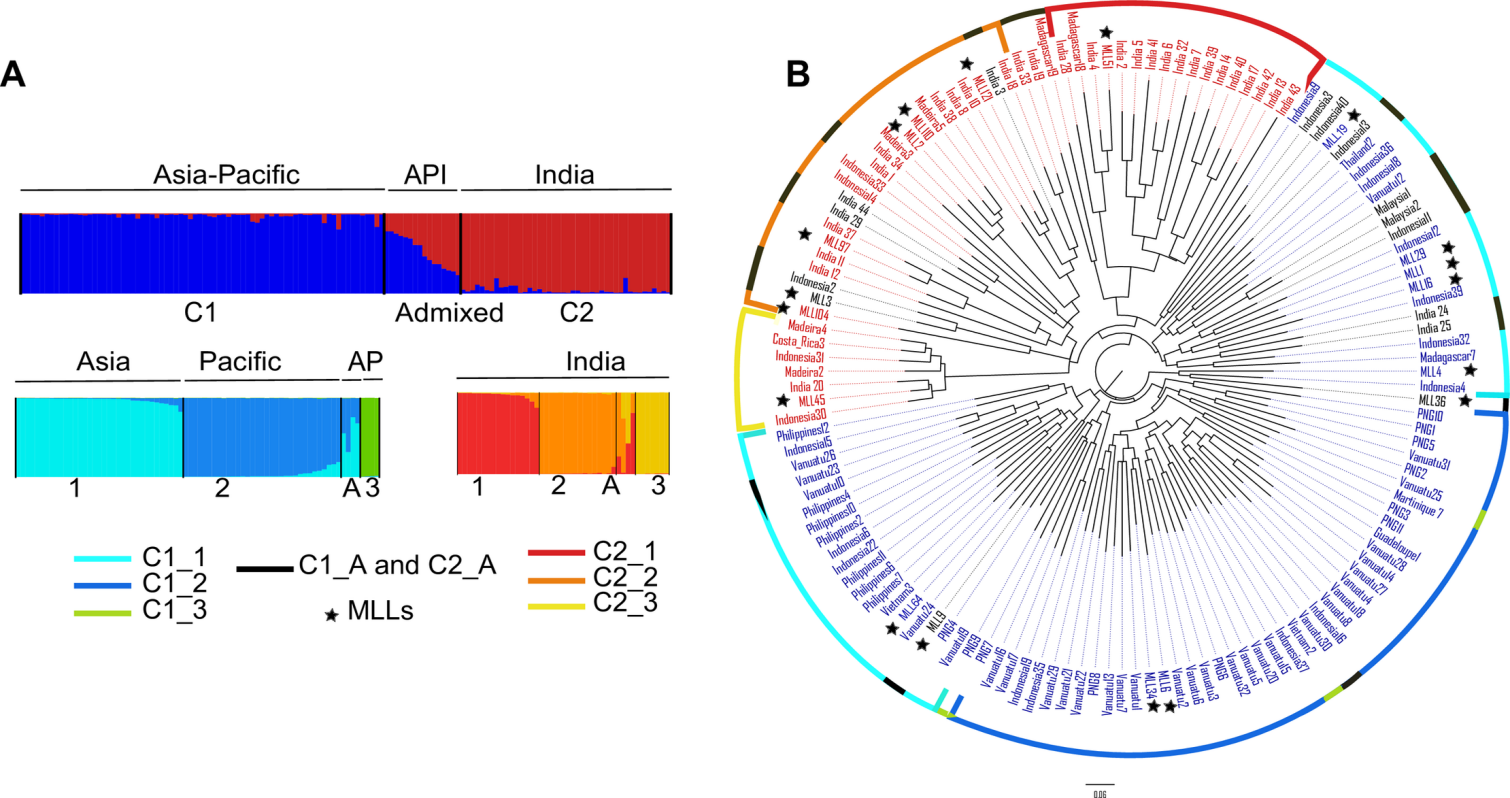
Genetic relationships and Genetic structure of the 136 genotypes (UGs and MLLs). (A) Unrooted neighbor-joining tree, based on 11 microsatellite markers, using Dice distance, showing genetic relationships among 136 genotypes. Each node label is colour-coded according to membership in the two clusters C1 and C2 identified by STRUCTURE. Genotypes assigned to admixed groups are shown in black. Outer circles are colour-coded according to sub-clustering within Clusters 1 and 2. Genotypes assigned to admixed groups after sub-clustering are shown in black. (B) Cluster assignment of 136 taro genotypes estimated using STRUCTURE for K = 2 and sub-cluster within each cluster for K = 3. The genome of each individual is represented by a vertical line, which is partitioned into K colored segments that represent the admixture coefficient, i.e the estimated proportion of membership of its genome in each of the K clusters. API: Genotypes from Asia, Pacific and India. AP: Genotypes from Asia and Pacific. 1, 2, 3 and A: Sub-clusters and admixed genotypes within each cluster C1 and C2.

To investigate population structuring, a Bayesian population structure analysis was first performed on the whole identified MLLs and UGs dataset, representing 136 genotypes. The log likelihood increased from K = 1 to K = 10. Model selection based on Δ*K* supported K = 2 as a possible value for the uppermost structure level. Using K = 2, 120 genotypes had more than 80% membership in one cluster (Table 3, Fig 2A).

**Table 3:** Distribution of the cultivars in Clusters 1 and 2 and in the sub-clusters after STRUCTURE analysis.

	Clusters	C1: Asia-Pacific				A		C2: India			
	Sub-Clusters	C1_1	C1_2	C1_3	C1_A			C2_1	C2_2	C2_3	C2_A
Continent	Countries	Asia	Pacific1	Pacific2				India1	India2	India3	
Africa	Burkina Faso_Ghana (MLL2)							**33**			
	Madagascar	1							**1**		**1**
	Madeira							1/**1**		**2**	
America	Costa Rica									**1**	
	Guadeloupe		1								
	Martinique			1							
	Guadeloupe_Martinique (MLL6)		5								
	Guadeloupe Martinique Trinidad & Tobago (MLL34)		7								
Asia	India (UG + MLL97 + MLL121)					2	**4**	2/**6+6+2**	8/**6**	**1**	**3**
	India_Indonesia (MLL51)								**2**		
	India(2X)_Indonesia (3X) (MLL45)									1/**1**	
	Indonesia (UG+MLL19)	12+2	1	1		3	**1**	2		1	
	Indonesia_Philippines (MLL1)	4									
	Indonesia_Thailand (MLL9)					4					
	Indonesia (MLL16)	2									
	Malaysia					2					
	Philippines (UG+MLL64)	7+2									
	Thailand	1				1					
	Vietnam	1			1						
Pacific	Vanuatu	6	21	1	2						
	PNG	1	8	1	1						
Shared MLL between continents	Japan_South Africa (MLL104)							**5**			
	Indonesia_Madagascar (MLL36)					3					
	India_Nigeria(MLL110)							**2**			
	Costa Rica Ghana Japan Madeira Réunion South Africa Vietnam (MLL3)						**60**				
	Burkina Faso_Costa Rica_Ghana_Madagascar _Philippines_Réunion (MLL4)	96									
	Indonesia_Vanuatu (MLL29)	2									
**Total**		35	33	4	4		16	17	16	7	4

C1 and C2, main clusters obtained after STRUCTURE analysis. Admixed genotypes with membership of less than 80% in one of the clusters identified. C1_1, C1_2, C1_3 and C2_1, C2_2, C2_3, are the three sub-clusters identified within C1 and C2, respectively. C1_A and C2_A, admixed genotypes within C1 and C2, respectively. Triploid cultivars are in bold.

Of the 120 genotypes, 76 were grouped in the first cluster (C1), eight MLLs, including the widespread MLL4, and 68 UGs from 12 countries, representing genotypes which showed 2 alleles at all loci, including TANSAO diploids. They corresponded to all cultivars from the Pacific (i.e. PNG and Vanuatu), all cultivars from Asia (except India), a few ones from Indonesia, all cultivars from Caribbean islands and one from Madagascar.

In the second cluster (C2), 44 genotypes were grouped, representing 29 genotypes with three alleles at at least one locus, and 15 genotypes with two alleles at all of the investigated loci. They represented seven MLLs and 37 UGs. India was represented by 26 UGs and 5 MLLs (MLL 97, 121, 51, 45 and 110) whether they were grouped within India or shared with Indonesia or Nigeria (21 triploids and 10 diploids). The other genotypes assigned to C2 represented 10 UGs from Madagascar, Madeira, Costa Rica and Indonesia and two MLLs (MLL2 and 104) shared between Burkina Faso and Ghana and between Japan and South Africa.

**Fig 3:**
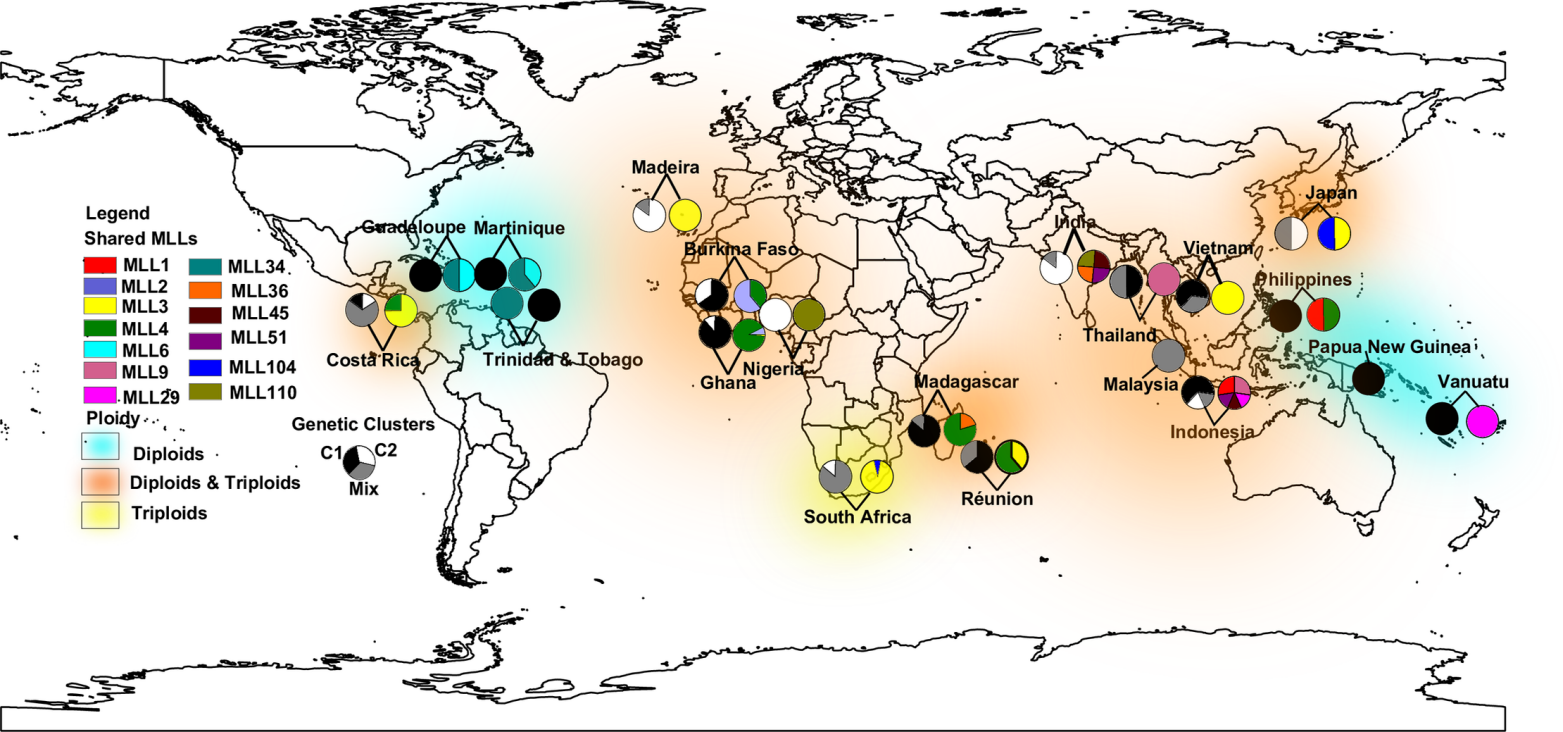
Map showing the geographical distribution of the cultivars (i) after Bayesian clustering assignment (black, grey and white pie chart in each linked pair of charts), (ii) of the ploidy levels inferred from the number of alleles per microsatellite locus, (iii) and of the multi-locus lineages (MLLs) in cultivated taro (coloured pie chart in each linked pair of charts).

The 16 genotypes with membership under 80% were admixed and corresponded to 10 diploids (including seven assessed by flow cytometry [1]) and six triploids (including four Indian cultivars assessed by chromosome counting). They represented 10 UGs, two MLLs (9 and 36) shared between Indonesia and Thailand and between Indonesia and Madagascar, and the widespread MLL 3 found in Costa Rica, Ghana, Japan, Madeira, Réunion, South Africa and Vietnam (Fig 3).

The Dice distance-based unrooted NJ tree showed groups consistent with the clusters obtained by STRUCTURE. The first group encompassed all MLLs and UGs assigned to cluster C1, while the second encompassed the ones assigned to cluster C2 (Fig 2B). Analyses of genetic parameters between the two clusters (C1 and C2) revealed that the number of private alleles, Shannon index and genetic diversity were higher in the second cluster that mainly pooled Indian cultivars (S3 Table).

In order to identify any sub-clustering within each cluster, we separately ran new STRUCTURE analyses on each cluster. A K = 1 to K = 10 increase in the log likelihood was obtained for both analyzed clusters. The ΔK obtained allowed us to identify K = 3 for both clusters (Table 3, Fig 2A). Cluster 1 was subdivided into three sub-clusters and admixed genotypes. The first sub-cluster (C1_1) encompassed mainly genotypes corresponding to cultivars from Asia (Indonesia, the Philippines, Thailand and Vietnam), respectively one cultivar from Madagascar and PNG, the six breeding lines from Vanuatu, the MLLs 1, 16, 29 and the widespread MLL4. The second sub-cluster (C1_2) encompassed genotypes corresponding to 33 cultivars mainly from the Pacific (21 from Vanuatu and eight from PNG), two from Guadeloupe and Indonesia and two MLLs (6 and 34) shared between Guadeloupe and Martinique and between these two last islands and Trinidad & Tobago. The third sub-cluster encompassed only four genotypes (Vanuatu, Martinique, Indonesia and PNG). Four genotypes were assigned to admixed group representing two cultivars from Vanuatu, one from PNG and the last from Indonesia. The second cluster (C2) was also subdivided into three sub-clusters containing diploids and triploids with, however, no structuring according to ploidy level. Seventeen genotypes were assigned to the first sub-cluster (C2_1), including two diploids and six triploids from India, two diploids from Indonesia and the triploid MLLs (2, 97, 121, 104 and 110). Sixteen genotypes were assigned to the second sub-cluster (C2_2), representing mainly cultivars from India (eight diploids and six triploids), one from Madagascar and MLL51 shared between India and Indonesia. The third sub-cluster (C2_3) encompassed mainly triploids, i.e. two cultivars from Madeira, one from Costa Rica, one from India and MLL45 from Indonesia. Finally, four genotypes were assigned to the admixed group corresponding to triploid cultivars (three from India and one from Madagascar).

## Discussion

### Taro genetic diversity and ploidy

This is the first time that an investigation on taro diversity has been extended to Africa and America. Previous studies were conducted at country and regional levels [26, 33], or in Asia and Oceania [1, 23]. There is a scarcity of data from studies conducted outside the taro geographical centre of origin [30]. Here we investigated the genetic diversity of 19 Asian, Pacific, African and American countries. The genetic parameters revealed that the diversity was greater in Asia than in the Pacific, Africa and America. Asia had the highest number of private alleles, Shannon index and diversity. This is in accordance with the hypothesis that taro originated from the Indo-Malayan area [13, 50]. Moreover, within the Asian pool, India had the highest genetic diversity [25]. Diversity in the Pacific region was lower than in Asia, as reported in previous studies [1, 25]. It was also suggested, using isozymes and AFLPs, that taro was probably domesticated in New Guinea and then carried by Austronesians as they spread to Polynesian and Micronesian islands. As a result, all cultivars in the Pacific share a common and narrow genetic base [23]. Our results are in accordance with a common narrow genetic origin due to a bottleneck effect but not with domestication in New Guinea. Moreover, in the present study and except for the number of private alleles, the Shannon index and diversity findings did not differ between the Pacific, America and Africa. This result is not in accordance with the relative importance of taro in the Pacific compared to the two other continents, where it is embedded in diverse cultures as a result of its selection for a wide variety of uses. Taro is often viewed as intrinsic to cultural identity, as in Hawaii where indigenous people believe that it is an ancestor [7]. In Africa, genetic parameters indicated a very low genetic diversity, very slightly higher in Madagascar and Madeira than in South Africa, Ghana and Burkina Faso. The difference could be related to the number of clones introduced in each country. In America, genetic diversity was very low and only two private alleles were found in our sample. The fact that the number of cultivars analyzed in the three Caribbean islands and Costa Rica was very low might be related to the number of clones introduced or to the importance of taro in this area. While taro has been widely adopted in Africa [19], it is less important in America where tannia (*Xanthosoma sagittifolium*), originating from America, is more frequently cultivated. Consequently these countries do not host important taro diversity.

We could not assess the ploidy level of all cultivars analyzed. However, the number of alleles obtained with the 11 microsatellite marker loci perfectly matched the ploidy levels assessed by flow cytometry and the chromosome counts in cultivars from Burkina Faso, India and those from the TANSAO collection. All cultivars from the Pacific displayed two alleles at all loci, which is in accordance with their diploid level, as reported in previous studies [1, 12, 23]. Both diploids and triploids were found on other continents. In the Caribbean islands (Guadeloupe, Martinique and Trinidad & Tobago) only diploids were found, while Costa Rica and South Africa hosted only triploid cultivars. This distribution was probably related to the different origins of taro introduced in these countries or to a selective adaptation to local environmental conditions.

### Taro diversification

The highest indices of clonal diversity were obtained in countries from Asia and the Pacific. Within these countries, it seems more likely that sexual reproduction was the means by which taro genetically diversified. In the Pacific, where all cultivars are diploids, many cultivars flower naturally, insect pollinators are very active, and natural hybridization among cultivars occurs regularly [4], so the clonal richness index is very high even though the genetic diversity is narrow. This has been reported in Vanuatu where 209 taro cultivars collected from six villages located on different islands had a clonal richness index of 0.83 [44]. In Africa, the clonal richness was not similar among countries analyzed and two groups could be distinguished: Réunion and Madagascar where the number of cultivars was low but, the clonal richness index between 0.3 and 0.5. In Madeira, located on the rim of the African continent, the clonal richness index was 1. This diversity was not related to sexual reproduction, because the number of triploids was very high, but more likely to better management and conservation of local cultivars. In the second group, represented by South Africa, Ghana and Burkina Faso, the clonal richness index was very low. In these countries, the few local cultivars could have been introduced from areas where the diversity was already low. Alternatively, a larger number of cultivars may have been introduced but, due to farmers’ selection and local environmental, only a few cultivars were maintained and disseminated in these countries. Finally, on the American continent, the clonal richness index is relatively high in Costa Rica and on the Caribbean islands, except Martinique (R = 0.22). The number of cultivars analyzed was very low but they were considered as representative of the cultivars grown in these countries, where other root and tuber crops are more appreciated, such as cassava, yam and tannia. In Guadeloupe, for example, the total cropping area has dramatically decreased, from 500–600 ha in the 1950s to less than 100 ha in 2003, with seven cultivars grown [5]. Hence, even though greater diversity was introduced in these countries, it seems that it was lost due to the abandonment of this crop to the benefit of others.

### Taro genetic structuring

Our Bayesian clustering analysis showed a clear separation between diploids mainly from the Asian-Pacific region and diploids and triploids mainly from India. This is the first time that such divergence between the two genetic pools has been clearly revealed. Most previous studies did not include Indian germplasm in their sampling [1, 25], or the number of samples considered was insufficient [23]. Other studies concerned only Indian germplasm and revealed high genetic diversity [28]. Consequently, the contribution of the Indian genetic pool to the worldwide taro diversity and evolution remained unclear. In our study, genetic diversity and the number of private alleles were higher in Indian cultivars than in Asian-Pacific cultivars. This high divergence led to two hypotheses: i) taro was domesticated in India and spread later towards the Asia-Pacific region, thus the two gene pools could have diverged later as a result of isolation by distance or ii) taro was domesticated independently in two areas, i.e. in the Asia-Pacific region and in India.

Most previous studies have assigned the diploids to the Pacific pool while the triploids were grouped in the Asian pool. In our study, while the Asian-Pacific pool encompassed most diploids from Asia and the Pacific, the Indian pool encompassed all diploid and triploid Indian cultivars and only four other diploid cultivars (three from Indonesia and one from Madeira). The remaining triploids were admixed between the Asian-Pacific and Indian genetic pools. It seems that all triploids arose from the Indian pool or were hybrids between the Indian and Asian-Pacific genetic pools and subsequently spread to other countries.

The two main sub-clusters found in the Asian-Pacific pool corresponded to the Asian and Pacific genetic groups reported in previous studies [1, 23, 25]. This is in accordance with the hypothesis of a secondary domestication in New Guinea [4]. Some discrepancies however were reported. Six cultivars from Vanuatu, representing the breeding lines obtained recently from Asia, and one from PNG were found in the Asian pool and one cultivar from Indonesia was found in the Pacific pool. This is due to the fact that many cultivars were recently exchanged between the two areas within the framework of the TANSAO project [1]. We found a third group encompassing two cultivars from the Pacific, one from Indonesia and one from Martinique. This group might correspond to another genetic group which has not been detected in previous studies due to the markers (isozyme, AFLP) and data analysis method used. No difference in corm quality or ecology has been reported for these cultivars. Due to the movement of crops in this area, especially roots and tubers [15, 26, 51], the geographical origin of these cultivars could have been lost. The Indian group was also subdivided into three sub-groups. The sub-clustering did not correspond to the ploidy level which suggests that the triploids were very close to the diploids. This finding supports the hypothesis that triploid taros have evolved from diploids and are of autopolyploid nature [52]. An additional set of chromosomes is considered to provide triploids with increased levels of adaptability and hardiness at high elevation and latitude [52]. While most of the Indonesian cultivars were assigned to the Asian group, three diploids and one triploid were assigned to the Indian group. Whether these cultivars originated from Indonesia or India and were brought to Indonesia remains unclear. Like the first Asian-Pacific group, the Indian one exhibited a third sub-group with a few cultivars corresponding to one Indian, two Indonesian and one MLL shared between India and Indonesia, one from Costa Rica and two from Madeira. Further sampling is necessary to trace the origin of this third sub-group.

### Taro dispersal to Africa and America

*Colocasia esculenta* is not native to Africa or America and has reached these two continents through human migration. Within our sample, MLL3 and MLL4 were shared between countries from Asia, Africa and Central America. These two MLLs represented nearly a third of the cultivars (156) and corresponded to 22 MLGs. It is unlikely that a single clone was introduced to different countries on different continents directly from a single point of origin. A gradual diffusion process is more likely, with a single clonal genotype spreading from one country to another in multiple directions from its point of origin as a seedling. During this dissemination process, it probably accumulated mutations leading to different MLGs. Our data suggest that both MLLs are most likely ancient introductions from Asia and/or India. After settlement, they were exchanged between Africa and America. Indeed, vegetative propagation is reported to enable the maintenance and spread of superior individuals but also to decrease the number of sexual cycles [53]. Consequently, superior genotypes are propagated clonally and spread by a mix of human migrations and material exchange. It might be the case for both of these MLLs.

All cultivars from West Africa and from Madeira were assigned to the Indian and Asian groups, except for MLL3 which was shared with other countries and assigned to an admixed genotype between the Asian-Pacific and Indian genetic groups. So it seems likely that taro in West Africa originated from India or other Asian countries rather than from the Pacific. It remains unclear how taro reached West Africa [19]. It seems likely that the history of taro introduction in West African countries and Madeira is not the same. Taro was supposed to have been introduced in Africa concomitantly with the bananas and the greater yam (*Dioscorea alata* L.) [19]. This “vegeculture trio” had probably reached the continent through Indian Ocean during the Iron Age as attested by the presence of banana phytoliths in pits in Cameroon dating of the mid-first millennium BCE [54]. While in Madeira, the Indian origin of taro could likely be explained by the fact that Madeira was on the road of Portuguese traders heading to spice sources in India. They might have brought back taro during that period of intense navigation between the 14th and 15th centuries. This crop is also found in the wild or cultivated on other Macaronesian islands (the Azores, Cape Verde and Canaries islands) that were important bases during the Colombian Exchange between the 15th and 16th centuries [29, 55]. Cultivars from Madagascar were assigned to both Asian and Indian subgroups and one MLL was shared with Indonesia. This is in agreement with the history of crop introductions and linguistic data in Madagascar [56]. Taro was identified as being among the first plants introduced by Austronesian who settled in Madagascar during the first Millennium CE. According to Portères [57], the local name *sonjo* has the same name root as the *sune* from Polynesia. The name *taho* is also found in Madagascar but in geographically localized areas and this term is thought to be related to Timor languages [56]. Indian germplasm could have arrived in Madagascar via Austronesian settlers since Indian MLLs are found in Indonesia, or via Portuguese traders as was probably the case in Madeira, or more recently via Indian settlers [58]. The same applies to Réunion, for which cultivars were assigned to MLL4 of Asian subgroup origin, shared with Madagascar, indicating the same history of plant introduction or intensive crop exchange between the two neighboring islands. Both MLLs present in South Africa were shared with Japan and were assigned to Indian and admixed between Indian and Asia-Pacific genetic groups. Taro is not native to Japan [59]. As MLL104 was not found in countries other than South Africa and Japan, it could have been introduced directly from Japan as trade relations between both countries were reported since 1643 [60], or it could have been introduced from an other Asian country.

In the present study, few cultivars were obtained from Central America and the Caribbean islands, and few countries were represented in the American sample. One MLL was shared between Guadeloupe, Martinique and Trinidad & Tobago; another was only shared between the first two, which is evidence of crops exchanges between Caribbean islands. All cultivars from Caribbean islands were assigned to the Pacific sub-group. Taro is also called *Madère* in Guadeloupe and Martinique which could suggest an introduction from Madeira. However, no accessions were grouped with the Madeira, i.e. Asian or Indian, gene pool. This could likely be explained by the fact that very few cultivars were analyzed. However, most present cultivars were in our sampling. Costa Rica was the only Central American country represented in our sampling, encompassing MLL 3 and 4 and one cultivar assigned to the third Indian small subgroup. MLL 3 and 4 were shared between Madeira, Japan, the Philippines and Vietnam, and also Madagascar and Réunion. Manzano et al. [29], in an isozyme analysis of Cuban taro cultivars, hypothesized that taro in Central America, could have been introduced directly from Japan or from the Canary islands, or introduced to Mexico from the Philippines via the Manila to Acapulco Spanish trade route, and then exchanged between the different neighboring countries. Thus, the introduction of taro in Central America remains unclear.

### Conclusion

Most diversity in taro has arisen through breeding, which has given rise to a large number of genotypes, as shown in the present study. Although taro is always propagated asexually in farmers' fields, it is an allogamous, highly heterozygous species, and natural pollinations do occur between flowering diploids. New genotypes germinating spontaneously can be clonally selected by farmers but these can also capture somaclonal variants when these appear sufficiently different form mother plants to be considered as new cultivars and renamed. Some of the genetic diversity described here reflects mutation over long periods of time in geographically widespread clonal lineages that may be very ancient. Some clonal lineages in taro have been widely distributed through migration or exchange. Further study of these widespread clones is needed to determine their geographical origins, antiquity, and likely routes of dispersal. However, taro being essential for food security in many developing countries, it appears necessary to broaden the genetic bases to facilitate farmers' varietal portfolios adaptation to climatic changes.

Most of the cultivars investigated in our study could be assigned to a genetic pool, so their region or continent of origin could thus be identified. We could not, however, determine the countries from which they originated or their dispersal routes. Further studies should focus on broadening sampling from Central Asia and East Africa and on including wild accessions and herbaria specimen vouchers. The use of uni-parentally inherited molecular markers (e.g. chloroplasts) will contribute to better identification of the geographical origins and to track taro dispersal routes through countries and continents. These studies should be carried out by combining genetic, archeological and historical data.

## Supporting Information

S1 Fig Unrooted neighbor-joining tree based on 11 microsatellite markers using Dice distance implemented in the Darwin V5 program, showing genetic relationships among 357 taro cultivars in the countries. Each branch is colour-coded according to the variety country of origin.
(PDF)

S2 Fig Genetic distances distribution frequencies for taro (*Colocasia esculenta* L.) Schott). Cultivars collected in 19 countries show bimodal distribution, calculated using Genotype software, with a small peak ranging from d = 0 (clonemates) to d = 8. The clonal threshold distance corresponds to the maximum distance below which distinct MLGs belong to the same clone is equal to d = 8.
(DOCX)

S1 Table Description of the 357 cultivars used to investigate taro diversity and dispersal using 11 nuclear microsatellite markers. Cultivars, cultivars names provided by each country and by SPC. Country of origin, the country where the cultivar was collected. Max number of alleles, Maximum number of alleles for the eleven loci analyzed. Assessed Ploidy Level, the ploidy level assessed by chromosome counting or flow cytometry. MLG, multilocus genotype after Genotype analysis. MLL_threshold 8, the assignment of the cultivars to MLL or UG. Genotypes_STRUCTURE, the selected genotypes, i.e. all UG and one genotype per MLL, used for Bayesian structure analysis. Structure_K2_q_0.8, is the assignation of each genotype to Clusters 1, 2 or admixed after Bayesian structure analysis.
(XLSX)

S2 Table Characteristics of the 11 primers used for genotyping the 357 *Colocasia esculenta* cultivars. Locus name, Repeat motif, Ta, Minimum and Maximum allele sizes (bp), Number of alleles obtained within our sample and authors.
(XLSX)

S3 Table Genetic diversity parameters of the two clusters identified within the 136 genotypes after Bayesian clustering analysis with STRUCTURE. *N*, number of genotypes within each cluster. *A*_*n*_, total number of alleles, *A*_*e’*_, number of effective alleles, *A*_*P*_, number of private alleles, *I*, Shannon's information index, *μh*, unbiased diversity.
(XLSX)

## References

[1] Kreike CM, Van Eck HJ, Lebot V. Genetic diversity of taro, *Colocasia esculenta* (L.) Schott, in Southeast Asia and the Pacific. Theor Applied Genet. 2004;109(4):761–8. 15156282 10.1007/s00122-004-1691-z

[2] Jianchu X, Yongping Y, Yingdong P, Ayad WG, Eyzaguirre PB. Genetic diversity in taro (*Colocasia esculenta* Schott, Araceae) in China: An ethnobotanical and genetic approach. Econ Bot. 2001;55(1):14–31.

[3] Attri BL, Singh DB, Singh C. Potential of new horticultural crops for food and nutritional security and soil conservation in Bay islands. Indian Forester. 2013;139(5):452–8.

[4] Lebot V. Tropical root and tuber crops: cassava, sweet potato, yams, aroids CABI; 2009.

[5] Saur É, Imbert D. Traditional taro monoculture in the swamp forest of Guadeloupe. Bois et Forêts des Tropiques. 2003;277(3):85–9.

[6] Hunt HV, Moots HM, Matthews PJ. Genetic data confirms field evidence for natural breeding in a wild taro population (*Colocasia esculenta*) in northern Queensland, Australia. Gen Resour Crop Evol. 2013;60(5):1695–707.

[7] Ramanatha RV, Matthews PJ, Eyzaguirre PB, Hunter D. The Global Diversity of Taro: Ethnobotany and Conservation. Ramanatha Rao V. MPJ, Eyzaguirre Pablo B., Hunter D, editor. Bioversity International, Rome, Italy 2010. 202 p.

[8] Loy TH, Spriggs M, Wickler S. Direct evidence for human use of plants 28,000 years ago-starch residues on stone artifacts from the northern Solomon-Islands. Antiquity. 1992;66(253):898–912.

[9] Fullagar R, Field J, Denham T, Lentfer C. Early and mid Holocene tool-use and processing of taro (*Colocasia esculenta*), yam (*Dioscorea sp*.) and other plants at Kuk Swamp in the highlands of Papua New Guinea. J Archaeol Sci. 2006;33(5):595–614.

[10] Horrocks M, Nunn PD. Evidence for introduced taro (*Colocasia esculenta*) and lesser yam (*Dioscorea esculenta*) in Lapita-era (c. 3050–2500 cal. yr BP) deposits from Bourewa, southwest Viti Levu Island, Fiji. J Archaeol Sci. 2007;34(5):739–48.

[11] Kuruvilla KM, Singh A. Karyotypic and electrophoretic studies on taro and its origin. Euphytica. 1981;30(2):405–13.

[12] Coates DJ, Yen DE, Gaffey PM. Chromosome variation in taro, *Colocasia esculenta*, implications for origin in the Pacific. Cytologia. 1988;53(3):551–60.

[13] Matthews P. A possible tropical wildtype taro: *Colocasia esculenta* var. *aquatilis*. Indo-Pacific Prehistory Assn Bulletin. 1991;11:69–81.

[14] Roullier C, Benoit L, McKey DB, Lebot V. Historical collections reveal patterns of diffusion of sweet potato in Oceania obscured by modern plant movements and recombination. Proc Natl Acad Sci. 2013;110(6):2205–10. 10.1073/pnas.1211049110 23341603 PMC3568323

[15] Perrier X, De Langhe E, Donohue M, Lentfer C, Vrydaghs L, Bakry F, et al Multidisciplinary perspectives on banana (*Musa spp*.) domestication. Proc Natl Acad Sci. 2011;108(28):11311–8. 10.1073/pnas.1102001108 21730145 PMC3136277

[16] Sanou H, Angulo-Escalante MA, Martinez-Herrera J, Kone S, Nikiema A, Kalinganire A, et al Loss of Genetic Diversity of *Jatropha curcas* L. through Domestication: Implications for Its Genetic Improvement. Crop Sci. 2015;55(2):749–59.

[17] Ndaeyo NU, Ekpe EO, Edem SO, Umoh UG. Growth and yield responses of *Colocasia esculenta* and *Xanthosoma saggitifolium* to tillage practices in Uyo, south-eastern Nigeria. Indian J Agricul Sci. 2003;73(4):194–8.

[18] Sanou J, Bayala J, Teklehaimanot Z, Bazie P. Effect of shading by baobab (*Adansonia digitata*) and nere (*Parkia biglobosa*) on yields of millet (*Pennisetum glaucum*) and taro (*Colocasia esculenta*) in parkland systems in Burkina Faso, West Africa. Agrof Syst. 2012;85(3):431–41.

[19] Blench R. Bananas and plantains in Africa: re-interpreting the linguistic evidence. Ethnobotany Research & Applications. 2009;7:363–80.

[20] Duss RP. Flore phanérogamique des Antilles françaises. II tIe, editor. Fort-de-France: Réédité en 1972 par la Société de Distribution et de Culture; 1897.

[21] Burlakova LE, Karatayev AY, Padilla DK, Cartwright LD, Hollas DN. Wetland Restoration and Invasive Species: Apple snail (*Pomacea insularum*) Feeding on Native and Invasive Aquatic Plants. Restor Ecol. 2009;17(3):433–40.

[22] Isshiki S, Otsuka K, Tashiro Y, Miyazaki S. A probable origin of triploids in taro *Colocasia esculenta* (L.) Schott. Journal of the Japanese Society for Horticultural Science. 1999;68(4):774–9.

[23] Lebot V, Aradhya KM. Isozyme variation in taro (*Colocasia esculenta* (L.) Schott) from Asia and Oceania. Euphytica. 1991;56(1):55–66.

[24] Irwin SV, Kaufusi P, Banks K, de la Pena R, Cho JJ. Molecular characterization of taro (*Colocasia esculenta*) using RAPD markers. Euphytica. 1998;99(3):183–9.

[25] Lebot V, Prana MS, Kreike N, van Heck H, Pardales J, Okpul T, et al Characterisation of taro (*Colocasia esculenta* (L.) Schott) genetic resources in Southeast Asia and Oceania. Genet Resour Crop Evol. 2004;51(4):381–92.

[26] Sardos J, Noyer J-L, Malapa R, Bouchet S, Lebot V. Genetic diversity of taro (*Colocasia esculenta* (L.) Schott) in Vanuatu (Oceania): an appraisal of the distribution of allelic diversity (DAD) with SSR markers. Genet Resour Crop Evol. 2012;59(5):805–20.

[27] Singh D, Mace ES, Godwin ID, Mathur PN, Okpul T, Taylor M, et al Assessment and rationalization of genetic diversity of Papua New Guinea taro (*Colocasia esculenta*) using SSR DNA fingerprinting. Genet Resour Crop Evol. 2008;55(6):811–22.

[28] Lakhanpaul S, Velayudhan KC, Bhat KV. Analysis of genetic diversity in Indian taro *Colocasia esculenta* (L.) Schott using random amplified polymorphic DNA (RAPD) markers. Genet Resour Crop Evol. 2003;50(6):603–9.

[29] Manzano AR, Nodals AAR, Gutiérrez MIR, Mayor ZF, Alfonso LC. Morphological and isoenzyme variability of taro (*Colocasia esculenta* L. Schott) germplasm in Cuba. Plant Genet Resour Newsletter. 2001;126:31–40.

[30] Nunes RSC, Pinhati FR, Golinelli LP, Reboucas TNH, Paschoalin VMF, da Silva JT. Polymorphic microsatellites of analysis in cultivars of taro. 41o Congresso Brasileiro de Olericultura I Encontro sobre plantas medicinais, aromaticas e condimentares, Brasilia—DF, Brazil, 22 a 27 de julho de 2001. 2012;30(1):106–11.

[31] Risterucci AM, Grivet L, N'Goran JAK, Pieretti I, Flament MH, Lanaud C. A high-density linkage map of *Theobroma cacao* L. Theor Appl Genet. 2000;101(5–6):948–55. 10.1007/BF00223910 24169987

[32] Hu K, Huang XF, Ke WD, Ding Y. Characterization of 11 new microsatellite loci in taro (*Colocasia esculenta*). Mol Ecol Resour. 2009;9(2):582–4. 10.1111/j.1755-0998.2008.02441.x 21564697

[33] Mace ES, Godwin ID. Development and characterization of polymorphic microsatellite markers in taro (*Colocasia esculenta*). Genome. 2002;45(5):823–32. 12416614 10.1139/g02-045

[34] Quero-Garcia J, Courtois B, Ivancic A, Letourmy P, Risterucci AM, Noyer JL, et al First genetic maps and QTL studies of yield traits of taro (*Colocasia esculenta* (L.) Schott). Euphytica. 2006;151(2):187–99.

[35] Santosa E, Lian CL, Pisooksantivatana Y, Sugiyama N. Isolation and characterization of polymorphic microsatellite markers in *Amorphophallus paeoniifolius* (Dennst.) Nicolson, Araceae. Mol Ecol Notes. 2007;7(5):814–7.

[36] Schuelke M. An economic method for the fluorescent labeling of PCR fragments. Nat Biotechnol. 2000;18(2):233–4. 10657137 10.1038/72708

[37] Bruvo R, Michiels NK, D'Souza TG, Schulenburg H. A simple method for the calculation of microsatellite genotype distances irrespective of ploidy level. Mol Ecol. 2004;13(7):2101–6. 15189230 10.1111/j.1365-294X.2004.02209.x

[38] Sampson JF, Byrne M. Genetic diversity and multiple origins of polyploid *Atriplex nummularia* Lindl. (Chenopodiaceae). Biol J Linnean Soc. 2012;105(1):218–30.

[39] Teixeira H, Rodriguez-Echeverria S, Nabais C. Genetic Diversity and Differentiation of *Juniperus thurifera* in Spain and Morocco as Determined by SSR. PLOS ONE. 2014;9(2). 10.1371/journal.pone.0088996 PMC3923062 24533164

[40] Peakall R, Smouse PE. GENALEX 6: genetic analysis in Excel. Population genetic software for teaching and research. Mol Ecol Notes. 2006;6(1):288–95. 10.1093/bioinformatics/bts460 PMC3463245 22820204

[41] Dice LR. Measures of the amount of ecologic association between species. Ecology. 1945;26(3):297–302.

[42] Perrier X, Jacquemoud-Collet J. DARwin software. 2006; Available: http://darwin.cirad.fr/darwin.

[43] Meirmans PG, Van Tienderen PH. GENOTYPE and GENODIVE: two programs for the analysis of genetic diversity of asexual organisms. Mol Ecol Notes. 2004;4(4):792–4.

[44] Vandenbroucke H, Mournet P, Vignes H, Chaïr H, Malapa R, Duval MF, et al Somaclonal variants of taro (*Colocasia esculenta* Schott) and yam (*Dioscorea alata* L.) are incorporated into farmers varietal portfolios in Vanuatu. Genet Resour Crop Evol. 2015:n.p. 26412939

[45] Arnaud-Haond S, Alberto F, Teixeira S, Procaccini G, Serrao EA, Duarte CM. Assessing genetic diversity in clonal organisms: Low diversity or low resolution? Combining power and cost efficiency in selecting markers. J Hered. 2005;96(4):434–40. 15743902 10.1093/jhered/esi043

[46] Scarcelli N, Tostain S, Vigouroux Y, Luong V, Baco MN, Agbangla C, et al Genetic structure of farmer-managed varieties in clonally-propagated crops. Genetica. 2011;139(8):1055–64. 10.1007/s10709-011-9607-8 21898046

[47] Pritchard JK, Stephens M, Donnelly P. Inference of population structure using multilocus genotype data. Genetics. 2000;155(2):945–59. 10835412 10.1093/genetics/155.2.945 PMC1461096

[48] Evanno G, Regnaut S, Goudet J. Detecting the number of clusters of individuals using the software STRUCTURE: a simulation study. Mol Ecol. 2005;14(8):2611–20. 15969739 10.1111/j.1365-294X.2005.02553.x

[49] Earl DA, vonHoldt BM. Structure Harvester: A website and program for visualizing STRUCTURE output and implementing the Evanno method. Conserv Genet Resour. 2012;4(2):359–61.

[50] Ivancic A, Lebot V. Botany and genetics of New Caledonian wild taro, *Colocasia esculenta*. Pacific Sci. 1999;53(3):273–85.

[51] Roullier C, Kambouo R, Paofa J, McKey D, Lebot V. On the origin of sweet potato (*Ipomoea batatas* (L.) Lam.) genetic diversity in New Guinea, a secondary centre of diversity. Heredity. 2013;110(6):594–604. 10.1038/hdy.2013.14 23531982 PMC3656641

[52] Ochiai T, Nguyen VX, Tahara M, Yoshino H. Geographical differentiation of Asian taro, *Colacasia esculenta* (L.) Schott, detected by RAPD and isozyme analyses. Euphytica. 2001;122(2):219–34.

[53] McKey D, Elias M, Pujol B, Duputie A. The evolutionary ecology of clonally propagated domesticated plants. New Phytologist. 2010;186(2):318–32. 10.1111/j.1469-8137.2010.03210.x 20202131

[54] Fuller DQ, Boivin N, Hoogervorst T, Allaby R. Across the Indian Ocean: the prehistoric movement of plants and animals. Antiquity. 2011;85(328):544–58.

[55] Gonçalves RF, Silva AMS, Silva AM, Valentão P, Ferreres F, Gil-Izquierdo A, et al Influence of taro (*Colocasia esculenta* L. Shott) growth conditions on the phenolic composition and biological properties. Food Chemistry. 2013;141(4):3480–5. 10.1016/j.foodchem.2013.06.009 23993510

[56] Beaujard P. The first migrants to Madagascar and their introduction of plants: linguistic and ethnological evidence. Azania. 2011;46(2):169–89.

[57] Portères R. La sombre Aroidée cultivée: *Colocasia Antiquorum* Schott ou taro de Polynésie. Essai d'étymologie sémantique. Journal d'agriculture tropicale et de botanique appliquée. 1960;7(4–5):169–92.

[58] Kusuma P, Cox MP, Pierron D, Razafindrazaka H, Brucato N, Tonasso L, et al Mitochondrial DNA and the Y chromosome suggest the settlement of Madagascar by Indonesian sea nomad populations. BMC Genomics. 2015;16(1):1394. 10.1186/s12864-015-1394-7 PMC4373124 25880430

[59] Matthews P, Matsushita Y, Sato T, Hirai M. Ribosomal and mitochondrial-DNA variation in japanese taro (*colocasia-esculenta* L. schott). Jap J Breed. 1992;42(4):825–33.

[60] Osada M. Sanctions and Honorary Whites: Diplomatic Policies and Economic Realities in Relations Between Japan and South Africa. United States of America: Greenwood Press; 2002. 255 p.

