# Supplementary figures and images for: Genetic Diversification and Dispersal of Taro (Colocasia esculenta (L.) Schott)
Source: PLoS One. 2016 Jun 17;11(6):e0157712. doi: 10.1371/journal.pone.0157712 (PMC4912093; doi:10.1371/journal.pone.0157712)

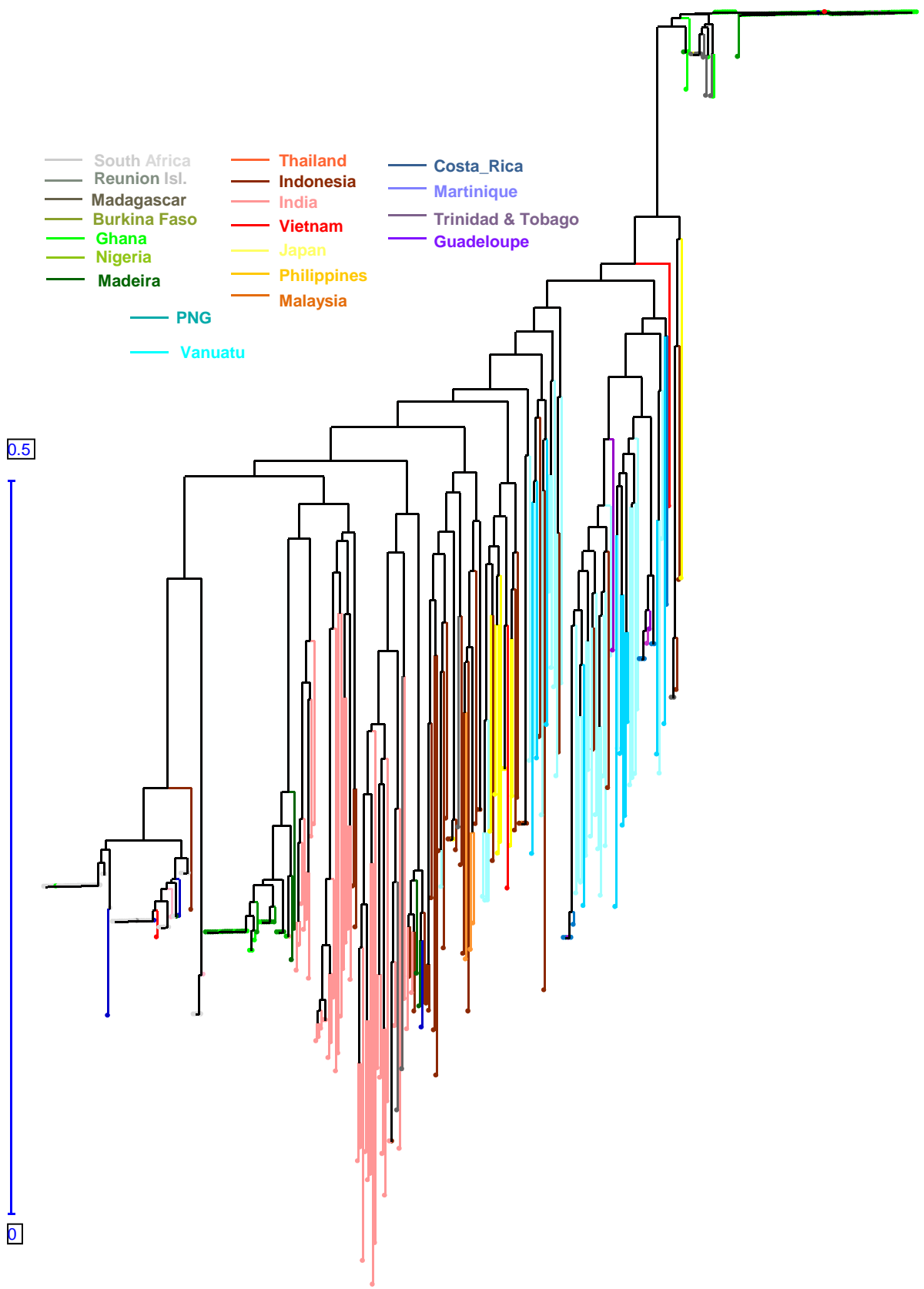

Supplement: S1 Fig — Each branch is colour-coded according to the variety country of origin. (PDF) [file pone.0157712.s001.pdf]
