# Supplementary material for: Genetic Diversification and Dispersal of Taro (Colocasia esculenta (L.) Schott)
Source: PLoS One. 2016 Jun 17;11(6):e0157712. doi: 10.1371/journal.pone.0157712 (PMC4912093; doi:10.1371/journal.pone.0157712)

**S2 Fig Genetic distances distribution frequencies for taro (*Colocasia esculenta*)**. Cultivars collected in 19 countries show bimodal distribution, obtained using Genotype software, with a small peak ranging from d = 0 (clonemates) to d = 8. The clonal threshold distance corresponds to the maximum distance below which distinct MLGs belong to the same clone is equal to d = 8.


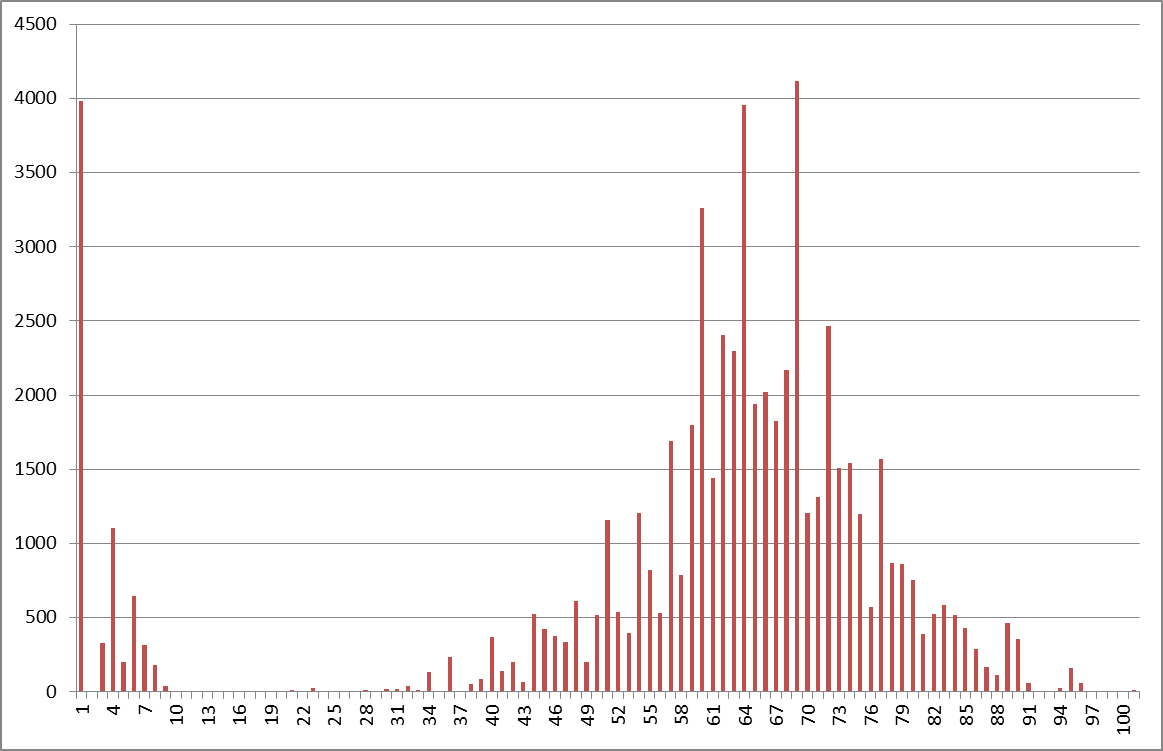

Supplement: S2 Fig — Cultivars collected in 19 countries show bimodal distribution, calculated using Genotype software, with a small peak ranging from d = 0 (clonemates) to d = 8. The clonal threshold distance corresponds to the maximum distance below which distinct MLGs belong to the same clone is equal to d = 8. (DOCX) [file pone.0157712.s002.docx]
